# Order Indices and Entanglement Production in Quantum Systems

**DOI:** 10.3390/e22050565

**Published:** 2020-05-18

**Authors:** Vyacheslav I. Yukalov

**Affiliations:** 1 Bogolubov Laboratory of Theoretical Physics, Joint Institute for Nuclear Research, Dubna 141980, Russia; yukalov@theor.jinr.ru; 2 Instituto de Fisica de São Carlos, Universidade de São Paulo, CP 369, São Carlos, São Paulo 13560-970, Brazil

**Keywords:** order indices, many-body systems, entanglement production, quantum operations, phase transitions, temporal evolution

## Abstract

The review is devoted to two important quantities characterizing many-body systems, order indices and the measure of entanglement production. Order indices describe the type of order distinguishing statistical systems. Contrary to the order parameters characterizing systems in the thermodynamic limit and describing long-range order, the order indices are applicable to finite systems and classify all types of orders, including long-range, mid-range, and short-range orders. The measure of entanglement production quantifies the amount of entanglement produced in a many-partite system by a quantum operation. Despite that the notions of order indices and entanglement production seem to be quite different, there is an intimate relation between them, which is emphasized in the review.

## 1. Introduction

Many-body systems can be described by different characteristics representing the main system properties. One of the pivotal notions is that of the type of order associated with the system state. The order is usually quantified by order parameters (see, e.g., [1,2,3]). The order parameters, as is well known, are rigorously defined in the thermodynamic limit, while for finite systems they, strictly speaking, degenerate to zero [3]. Here we mean a mathematically rigorous definition of order parameters, although in practical experiments and in numerical modeling one can still see the order parameters behave as if the thermodynamic limit is reached, when the system size is much larger than the correlation length.

At the same time, large many-body systems, even being finite, can posses a kind of order that is not of a long-range type, but rather it is a quasi-long-range or algebraic order. In addition, some two-dimensional systems exhibit quasi-long-range order below the Berezinskii–Kosterlitz–Thouless transition [4,5].

Order indices for density matrices were introduced in Refs. [6,7,8,9] as a general tool for quantifying all types of order, whether long-range, mid-range, or short-range. These characteristics can be defined for any statistical system, whether finite or in the thermodynamic limit. The notion of order indices can be generalized for arbitrary operators or matrices [10].

Another important notion characterizing the state of a multi-partite system is entanglement widely employed in quantum information processing and quantum computing [11,12,13,14,15,16,17], as well as in the theory of quantum measurements and quantum decision theory [18,19,20,21,22]. To be more precise, one has to distinguish between the entanglement of a state, or a statistical operator, or generally of an arbitrary operator, and the entanglement production describing the action of an operator on the given Hilbert space. The state entanglement characterizes the structure of a statistical operator, while the entanglement production by an operator describes the result of an operator action. These notions will be concretized below.

The aim of the present review is two-fold. First, a survey of the notions of order indices and of entanglement production, and their applications for treating many-body systems, will be given. Second, we shall emphasize the interrelation between these characteristics. It turns out that the latter are intimately related with each other, so that the qualitative change of a state leads to the quantitative changes of order indices as well as of the entanglement production.

Throughout the paper, the system of units is used where the Planck and Boltzmann constants are set to one.

## 2. Order Indices

Below, we consider systems composed of *N* parts, or particles, with *N* being finite, although it can be rather large. Suppose a collection $\left\{\hat{A}\right\}$ of trace-class operators acts on a Hilbert space H, so that

(1) $$0<|\;{\mathrm{Tr}}_{H}\hat{A}\;|<\infty \;.$$

The order index of an operator $\hat{A}$ is defined as

(2) $$\omega \left(\hat{A}\right)\equiv \frac{\log||\;\hat{A}\;\left|\right|}{\log|\;{\mathrm{Tr}}_{H}\hat{A}\;|}\;,$$

where the base of the logarithm can be taken according to convenience, since the above definition does not depend on the choice of the base due to the property $\log_{a}\left(x\right)=\log_{b}\left(x\right)\log_{a}\left(b\right)$. In other words, the order index is the exponent connecting the norm and trace of an operator,

$$||\;\hat{A}\;||=|\;{\mathrm{Tr}}_{H}\hat{A}{\;|}^{\omega (\hat{A})}\;.$$

In the case of a positive operator,

$$||\;\hat{A}\;\left|\right|\leq {\mathrm{Tr}}_{H}\hat{A}\;\left(\hat{A}\geq 0\right)\;,$$

because of which

(3) $$\omega \left(\hat{A}\right)\leq 1\;\left(\hat{A}\geq 0\right)\;.$$

As the norm, it is possible to take some of the Shatten norms

$$||\;\hat{A}\;{\left|\right|}_{p}\equiv {\left({\mathrm{Tr}}_{H}{\left|\;\hat{A}\;\right|}^{p}\right)}^{1/p}\;,$$

where

$$|\;\hat{A}\;|\equiv \sqrt{{\hat{A}}^{+}\hat{A}}\;\left(p\in \left[1,\infty \right]\right)\;.$$

Thus, for p=1, we have the trace norm

$$||\;\hat{A}{\;||}_{1}={\mathrm{Tr}}_{H}\left|\;\hat{A}\;\right|\;,$$

and for p=2, we come to the Hilbert–Shmidt norm

$$||\;\hat{A}\;{\left|\right|}_{2}={\left({\mathrm{Tr}}_{H}{\left|\;\hat{A}\;\right|}^{2}\right)}^{1/2}\;.$$

Below, it will be more convenient to accept the operator norm corresponding to p=∞, which gives the operator norm

(4) $$||\;\hat{A}\;{\left|\right|}_{\infty }=\underset{\varphi }{\sup}\;\frac{||\;\hat{A}\varphi \;\left|\right|}{\left||\;\varphi \;|\right|}\;\left(\varphi \neq 0\right)$$

generated by the vector norm

$$||\;\hat{A}\varphi \;\left|\right|=\sqrt{\left(\hat{A}\varphi ,\hat{A}\varphi \right)}\;.$$

Dealing with Hermitian operators, we get

(5) $$||\;\hat{A}\;{\left|\right|}_{\infty }=\underset{\varphi }{\sup}\;\frac{\left(\varphi ,\hat{A}\varphi \right)}{\left||\;\varphi \;|\right|}\;\left({\hat{A}}^{+}=\hat{A}\right)\;.$$

Comparing two operators ${\hat{A}}_{1}$ and ${\hat{A}}_{2}$, we say that the operator having a larger order index is better ordered. In physical applications, the operator or matrix order indices characterize the type of order associated with the considered operators. Examples will be given below.

## 3. Entangled Structures

One has to distinguish two different notions, entangled structures and entangling operations. The first notion characterizes the property of such structures as wave functions, statistical operators (quantum states), and which can be generalized to arbitrary operators. The second notion describes the action of quantum operations on the members of a Hilbert space. To better explain the difference of these notions, in the present section we recall the main definitions concerning entangled structures, and in the next section we shall elucidate the meaning of the entanglement production by quantum operations.

### 3.1. Entangled Functions

The notion of wave-function entanglement was introduced by Schrödinger [23,24] with respect to quantum systems that can be separated into several subsystems. Then the system Hilbert space can be represented as the tensor product

(6) $$H=\overset{n}{\underset{i=1}{⨂}}H_{i}$$

of the subsystem Hilbert spaces. Wave functions of the whole system, pertaining to the space H can be separated into two classes, separable and entangled functions. Separable functions have the form of the product

(7) $$\varphi_{sep}=\underset{i}{⨂}\varphi_{i}\;\left(\varphi_{i}\in H_{i}\right)\;.$$

Entangled functions can be represented as the linear combinations

(8) $$\varphi_{ent}=\sum_{\alpha }c_{\alpha }\underset{i}{⨂}\varphi_{i\alpha }\;\left(\varphi_{i\alpha }\in H_{i}\right)\;,$$

where at least two coefficients $c_{\alpha }$ are not zero. From the physical point of view, separable wave functions are rather exceptional, being attributed to quantum subsystems that have never interacted and are distinguishable [25]. Generally, subsystems are characterized by entangled wave functions [26,27].

The collection of all separable functions forms a disentangled set

(9) $$D=\{\varphi_{sep}\in H\}\;.$$

All entangled functions compose an entangled set

(10) $$H\setminus D=\{\varphi_{ent}\in H\}\;.$$

Considering wave functions, one keeps in mind the functions normalized to one, but in principle, the same definitions are valid for non-normalized functions that are the members of the Hilbert space H.

### 3.2. Entangled States

Generally, quantum states are represented by statistical operators. For pure states, statistical operators can be written as

(11) $$\hat{\rho }=|\;\varphi \;\rangle \langle \;\varphi \;|\;,$$

where the standard bra-ket notation is used. Separable wave functions compose separable states

(12) $${\hat{\rho }}_{sep}=|\;\varphi_{sep}\;\rangle \langle \;\varphi_{sep}\;|=\underset{i}{⨂}\;|\;\varphi_{i}\;\rangle \langle \;\varphi_{i}\;|\;,$$

while entangled wave functions form entangled states

(13) $${\hat{\rho }}_{ent}=|\;\varphi_{ent}\;\rangle \langle \;\varphi_{ent}\;|=\sum_{\alpha \beta }c_{\alpha }^{*}c_{\beta }\;\underset{i}{⨂}\;|\;\varphi_{i\beta }\;\rangle \langle \;\varphi_{i\alpha }\;|\;.$$

More generally, a state is separable if and only if it has the structure

(14) $${\hat{\rho }}_{sep}=\sum_{\alpha }\lambda_{\alpha }\;\underset{i}{⨂}{\hat{\rho }}_{i\alpha }\;,$$

in which

$$0\leq \lambda_{\alpha }\leq 1\;,\;\sum_{\alpha }\lambda_{\alpha }=1$$

and ${\hat{\rho }}_{i\alpha }$ is a statistical operator acting on a partial Hilbert space $H_{i}$. A state that cannot be represented in the above form is entangled.

### 3.3. Entangled Operators

The notion of entangled states can be generalized to trace-class entangled operators [19,20,21]. Let us consider an algebra $A=\{\hat{A}\}$ of trace-class operators acting on a Hilbert space $H_{A}$. For any two operators ${\hat{A}}_{1}$ and ${\hat{A}}_{2}$ from the algebra A one can introduce the Hilbert–Schmidt scalar product

$$\left({\hat{A}}_{1},\;{\hat{A}}_{2}\right)\equiv {\mathrm{Tr}}_{H}\left({\hat{A}}_{1}^{+}{\hat{A}}_{2}\right)\;.$$

The triple of the operator algebra A, acting on the Hilbert space $H_{A}$, and the Hilbert–Schmidt scalar product form the Hilbert–Schmidt space

$$\tilde{A}\equiv \left\{A,\;H_{A},\left(,\right)\right\}\;.$$

Similarly, one can define the Hilbert–Schmidt space for another trace-class operator algebra B as

$$\tilde{B}\equiv \left\{B,\;H_{B},\left(,\right)\right\}\;.$$

The composite Hilbert–Schmidt space is given by the tensor product

(15) $$\tilde{A}⨂\tilde{B}=\left\{A,\;H_{A},\left(,\right)\right\}⨂\left\{B,\;H_{B},\left(,\right)\right\}.$$

An operator ${\hat{C}}_{sep}$ from the composite Hilbert–Schmidt space in Equation (15) is separable if and only if it can be represented in the form

(16) $${\hat{C}}_{sep}=\sum_{\alpha }\lambda_{\alpha }{\hat{A}}_{\alpha }⨂{\hat{B}}_{\alpha }\;\left({\hat{A}}_{\alpha }\in \tilde{A},\;{\hat{B}}_{\alpha }\in \tilde{B}\right)\;.$$

Otherwise, the operator is entangled.

In general, an operator ${\hat{C}}_{sep}$, defined on the Hilbert space in Equation (6) is separable if and only if it can be represented as

(17) $${\hat{C}}_{sep}=\sum_{\alpha }\lambda_{\alpha }\underset{i}{⨂}{\hat{A}}_{i\alpha }\;,$$

where the operators ${\hat{A}}_{i\alpha }$ act on $H_{i}$.

These definitions lift the notion of entanglement to the operator level. In the Hilbert–Schmidt space, operators are isomorphic to the space members, so that the distinction between entangled and separable states in the Hilbert–Schmidt space becomes similar to that in the Hilbert space [19,20,21,28,29,30,31].

## 4. Entangling Operations

The other notion is the entanglement production by quantum operations. Considering the operators acting on a Hilbert space H, it is possible to distinguish two types of the operator actions, entangling and nonentangling. If an operator $\hat{A}$, acting on any function from the disentangled set D, leaves the function in this set, it is called a nonentangling operator [32,33],

(18) $$\hat{A}D\to D\;\left(\mathrm{nonentangling}\right)\;.$$

However, if there exists at least one function of the disentangled set D that becomes entangled under the action of the operator, this operator is termed entangling [34,35]. The strongest type of an entangling operator is a universal entangling operator that makes all disentangled functions entangled [36],

(19) $$\hat{A}D\to H\setminus D\;\left(\mathrm{entangling}\right)\;.$$

A principal problem is how to measure the entangling power of operators. When one is interested in just a few wave functions, it is admissible to analyze the action of a given operator on all the functions of interest. In the case of a bipartite system, one can check the amount of the produced entanglement by studying the entanglement entropy for the considered few wave functions [28,30,31,37]. This, however, does not allow for quantifying the entangling power of the examined operator on the whole Hilbert space.

A general measure of entanglement production for arbitrary trace-class operators acting on a Hilbert space was advanced in Refs. [38,39]. The idea behind the definition of this measure is to compare the action of the given operator $\hat{A}$ with the action of its nonentangling counterpart

(20) $${\hat{A}}^{⊗}\equiv \frac{{⨂}_{i=1}^{n}{\hat{A}}_{i}}{{\left({\mathrm{Tr}}_{H}\hat{A}\right)}^{n-1}}\;,$$

in which ${\hat{A}}_{i}$ are partially traced operators

(21) $${\hat{A}}_{i}\equiv {\mathrm{Tr}}_{H/H_{i}}\hat{A}\;.$$

The coefficient in Equation (20) is defined so that to preserve the trace normalization

(22) $${\mathrm{Tr}}_{H}\hat{A}={\mathrm{Tr}}_{H}{\hat{A}}^{⊗}\;.$$

The measure of entanglement production [38,39] by an operator $\hat{A}$ on a Hilbert space H is

(23) $$\epsilon (\hat{A})\equiv \log\;\frac{||\;\hat{A}\;\left|\right|}{||\;{\hat{A}}^{⊗}\;\left|\right|}\;.$$

This measure is based on the comparison of the action of an operator on the whole Hilbert space with the action of its nonentangling counterpart that leaves invariant the disentangled set,

$$\hat{A}H\to H\;,\;{\hat{A}}^{⊗}D\to D\;.$$

It is useful to emphasize that, as has been proved [40,41,42,43,44,45], the only operators preserving separability are the operators having the form of tensor products of local operators and a swap operator permuting Hilbert subspaces in the tensor product of the total Hilbert space of a composite system. However, the action of the swap operator is trivial, merely permuting the indices labeling the subspaces. Up to the enumeration of subspaces, the product operators are the sole operators preserving the separability of functions. The tensor-product operators, as is evident, do not produce entanglement,

$$\epsilon ({\hat{A}}^{⊗})=0\;.$$

Thus, the entanglement-production measure (23) is zero for nonentangling operators, and also it is continuous, additive, and invariant under local unitary operations [39,46]. As it should be for being a measure, it is semipositive. The sketch of the proof of this important property is as follows [39,46].

The set of trace-class operators $\hat{A}$ acting on the Hilbert space H, with a given operator norm, forms the Banach space

(24) $$B\left(\hat{A}\right)=\{\hat{A},\;H,\;||\;\hat{A}\;||\}\;,$$

which is a complete normed linear space. Similarly, the set of the product operators ${\hat{A}}^{⊗}$, leaving invariant the disentangled set D, composes the Banach space

(25) $$B\left({\hat{A}}^{⊗}\right)=\{{\hat{A}}^{⊗},\;D,\;||\;{\hat{A}}^{⊗}\;||\}\;.$$

The latter space, by definition, is a subspace of the Banach space seen in Equation (24),

$$B\left({\hat{A}}^{⊗}\right)\subset B\left(\hat{A}\right)\;.$$

Then it is admissible to define a projector transforming the members of space in Equation (24) into the members of space in Equation (25),

(26) $${\hat{P}}_{⊗}\hat{A}={\hat{A}}^{⊗}\;,$$

with the standard projector properties

$${\hat{P}}_{⊗}^{2}={\hat{P}}_{⊗}\;,\;{\hat{P}}_{⊗}^{+}={\hat{P}}_{⊗}\;,\;||\;{\hat{P}}_{⊗}\;\left|\right|=1\;.$$

Therefore we have

$$||\;{\hat{A}}^{⊗}\;||=||\;{\hat{P}}_{⊗}\hat{A}\;||\leq ||\;{\hat{P}}_{⊗}\;||\cdot ||\;\hat{A}\;||=||\;\hat{A}\;\left|\right|\;,$$

from where the semi-positivity of the measure follows:

(27) $$\epsilon (\hat{A})\geq 0\;.$$

It is important to stress that the entangled structure of an operator and entanglement production by this operator are quite different notions. An operator can be separable but entangling. Thus, the action of the separable operator in Equation (17) on the separable function in Equation (7) results in an entangled function,

$${\hat{C}}_{sep}\varphi_{sep}=\varphi_{ent}\;,$$

where

$$\varphi_{ent}=\sum_{\alpha }\lambda_{\alpha }\underset{i}{⨂}\varphi_{i\alpha }^{\prime }\;\left(\varphi_{i\alpha }^{\prime }\equiv {\hat{A}}_{i\alpha }\varphi_{i}\right)\;.$$

It is possible to notice that the measure of entanglement production in Equation (23) and the order indices in Equation (2), although having rather different meanings, but are connected with each other through the relations

$$\epsilon (\hat{A})=\log\;\frac{|\;{\mathrm{Tr}}_{H}\hat{A}{\;|}^{\omega (\hat{A})}}{||\;{\hat{A}}^{⊗}\;\left|\right|}\;,\;\omega \left(\hat{A}\right)=\frac{\epsilon (\hat{A})+\log||{\hat{A}}^{⊗}\left|\right|}{\log|{\mathrm{Tr}}_{H}\hat{A}|}\;.$$

The relations between these quantities will be considered in more detail below.

## 5. Density Matrices

To illustrate physical applications of the introduced notions, it is reasonable to consider such important physical quantities as reduced density matrices, which can be treated as matrix elements of reduced density operators [47]. For instance, the first-order density operator

(28) $${\hat{\rho }}_{1}=\left[\;\rho \left(x,x^{\prime }\right)\;\right]$$

is expressed through the matrix elements

(29) $$\rho \left(x,x^{\prime }\right)={\mathrm{Tr}}_{F}\psi \left(x\right)\;\hat{\rho }\;{\hat{\psi }}^{†}\left(x^{\prime }\right)=\langle \;\psi^{†}\left(x^{\prime }\right)\psi \left(x\right)\;\rangle \;.$$

Here *x* denotes a set of variables, such as spatial coordinates and spin, ψ(x) are field operators, the trace is over the Fock space generated by the field operators [48], and $\hat{\rho }$ is a statistical operator. The second-order density operator

(30) $${\hat{\rho }}_{2}=\left[\;\rho_{2}\left(x_{1},x_{2},x_{1}^{\prime },x_{2}^{\prime }\right)\;\right]$$

has the matrix elements

(31) $$\rho_{2}\left(x_{1},x_{2},x_{1}^{\prime },x_{2}^{\prime }\right)={\mathrm{Tr}}_{F}\psi \left(x_{1}\right)\psi \left(x_{2}\right)\;\hat{\rho }\;\psi^{†}\left(x_{2}^{\prime }\right)\psi^{†}\left(x_{1}^{\prime }\right)=\;=\langle \;\psi^{†}\left(x_{2}^{\prime }\right)\psi^{†}\left(x_{1}^{\prime }\right)\psi \left(x_{1}\right)\psi \left(x_{2}\right)\;\rangle \;.$$

Generally, the *n*-th order density operator is defined through the matrix elements

(32) $$\rho_{n}\left(x_{1},x_{2},\ldots ,x_{n},x_{1}^{\prime },x_{2}^{\prime },\ldots ,x_{n}^{\prime }\right)={\mathrm{Tr}}_{F}\psi \left(x_{1}\right)\psi \left(x_{2}\right)\ldots \psi \left(x_{n}\right)\;\hat{\rho }\;\psi^{†}\left(x_{n}^{\prime }\right)\ldots \psi^{†}\left(x_{2}^{\prime }\right)\psi^{†}\left(x_{1}^{\prime }\right)=\;=\langle \;\psi^{†}\left(x_{n}^{\prime }\right)\ldots \psi^{†}\left(x_{2}^{\prime }\right)\psi^{†}\left(x_{1}^{\prime }\right)\psi \left(x_{1}\right)\psi \left(x_{2}\right)\ldots \psi \left(x_{n}\right)\;\rangle \;.$$

The reduced density matrices of different orders are connected with each other by means of the relations

(33) $$\int \rho_{n}\left(x_{1},x_{2},\ldots ,x_{n},x_{1}^{\prime },x_{2}^{\prime },\ldots ,x_{n}\right)\;dx_{n}=\;=\left(N-n+1\right)\rho_{n-1}\left(x_{1},x_{2},\ldots ,x_{n-1},x_{1}^{\prime },x_{2}^{\prime },\ldots ,x_{n-1}^{\prime }\right)\;,$$

in which *N* is the number of particles in the system. For example,

(34) $$\int \rho_{2}\left(x_{1},x_{2},x_{1}^{\prime },x_{2}\right)\;dx_{2}=\left(N-1\right)\rho \left(x_{1},x_{1}^{\prime }\right)\;.$$

The trace operation here implies the summation over the variable *x*, defined as

(35) $$\mathrm{Tr}{\hat{\rho }}_{n}=\int \rho_{n}\left(x_{1},x_{2},\ldots ,x_{n},x_{1},x_{2},\ldots ,x_{n}\right)\;dx_{1}dx_{2}\ldots dx_{n}=\frac{N!}{(N-n)!}\;.$$

In particular

(36) $$\mathrm{Tr}{\hat{\rho }}_{1}=\int \rho \left(x,x\right)\;dx=N\;,\;\mathrm{Tr}{\hat{\rho }}_{2}=\int \rho_{2}\left(x_{1},x_{2},x_{1},x_{2}\right)\;dx_{1}dx_{2}=N\left(N-1\right)\;.$$

The non-entangling product operator for the second-order density matrix is expressed through the matrix elements

(37) $$\rho_{2}^{⊗}\left(x_{1},x_{2},x_{1}^{\prime },x_{2}^{\prime }\right)=\frac{N-1}{N}\;\rho \left(x_{1},x_{1}^{\prime }\right)\rho \left(x_{2},x_{2}^{\prime }\right)\;,$$

so that

(38) $$||\;{\hat{\rho }}_{2}^{⊗}\;||=\frac{N-1}{N}\;||\;{\hat{\rho }}_{1}\;{\left|\right|}^{2}\;.$$

Keeping in mind the operator norm in Equation (5), associated with the vector norm, we have

(39) $$||\;{\hat{\rho }}_{1}\;||=\underset{k}{\sup}\left(\varphi_{k},\;{\hat{\rho }}_{1}\varphi_{k}\right)\;(||\;\varphi_{k}\;||=1)\;,\;||\;{\hat{\rho }}_{2}\;\left|\right|=\underset{kp}{\sup}\left(\varphi_{k}\varphi_{p},\;{\hat{\rho }}_{2}\varphi_{k}\varphi_{p}\right)\;,$$

where $\left\{\varphi_{k}\right\}$ is a natural ortho-normalized basis [47] and *k* is a multi-index labeling quantum states. In first order, we have

(40) $$||\;{\hat{\rho }}_{1}\;\left|\right|=\underset{k}{\sup}N_{k}\;,$$

with the notation

$$N_{k}=\int \varphi_{k}^{*}\left(x\right)\rho \left(x,x^{\prime }\right)\varphi_{k}\left(x^{\prime }\right)\;dxdx^{\prime }\;.$$

In the second order, we find

(41) $$||\;{\hat{\rho }}_{2}\;\left|\right|=\underset{kp}{\sup}N_{kp}\;,$$

with

$$N_{kp}=\int \varphi_{k}^{*}\left(x_{1}\right)\varphi_{p}^{*}\left(x_{2}\right)\rho_{2}\left(x_{1},x_{2},x_{1}^{\prime },x_{2}^{\prime }\right)\varphi_{p}\left(x_{2}^{\prime }\right)\varphi_{k}\left(x_{1}^{\prime }\right)\;dx_{1}dx_{2}dx_{1}^{\prime }dx_{2}^{\prime }\;.$$

In this way, we can calculate the order indices of density matrices

(42) $$\omega \left(\rho_{n}\right)=\frac{\log||\;{\hat{\rho }}_{n}\;\left|\right|}{\log|\;\mathrm{Tr}{\hat{\rho }}_{n}\;|}$$

as well as the measure of entanglement production

(43) $$\epsilon ({\hat{\rho }}_{n})=\log\;\frac{||\;{\hat{\rho }}_{n}\;\left|\right|}{||\;{\hat{\rho }}_{n}^{⊗}\;\left|\right|}\;.$$

Using the properties of reduced density matrices, one can show [9,10,47,49] that the order indices, depending on statistics, satisfy the inequalities

(44) $$\omega \left({\hat{\rho }}_{n}\right)\leq 1\;\left(\mathrm{Bose}\right)$$

for Bose–Einstein statistics and

(45) $$\omega \left({\hat{\rho }}_{2n}\right)\leq \frac{1}{2}\;,\;\omega \left({\hat{\rho }}_{2n+1}\right)\leq \frac{n}{2n+1}\;\left(\mathrm{Fermi}\right)$$

for Fermi–Dirac statistics. When the upper boundary is reached, this signifies the occurrence of long-range order. Otherwise, there can only be mid-range or short-range order.

## 6. Correlation Matrices

Reduced density matrices are a particular case of correlation functions constructed by means of the field operators. In general, it is possible to consider correlation functions based on some other operators. Thus, taking an arbitrary operator $\hat{A}\left(x\right)$, acting on the Fock space, one can set the correlation functions

(46) $$C_{n}\left(x_{1},x_{2},\ldots ,x_{n},x_{1}^{\prime },x_{2}^{\prime },\ldots ,x_{n}^{\prime }\right)\equiv {\mathrm{Tr}}_{F}\hat{A}\left(x_{1}\right)\hat{A}\left(x_{2}\right)\ldots \hat{A}\left(x_{n}\right)\;\hat{\rho }\;\hat{A}\left(x_{n}^{\prime }\right)\ldots \hat{A}\left(x_{2}^{\prime }\right)\hat{A}\left(x_{1}^{\prime }\right)=\;=\langle \;\hat{A}\left(x_{n}^{\prime }\right)\ldots \hat{A}\left(x_{2}^{\prime }\right)\hat{A}\left(x_{1}^{\prime }\right)\hat{A}\left(x_{1}\right)\hat{A}\left(x_{2}\right)\ldots \hat{A}\left(x_{n}\right)\;\rangle \;.$$

Then, one can introduce the correlation operator

(47) $${\hat{C}}_{n}=\left[\;C_{n}\left(x_{1},x_{2},\ldots ,x_{n},x_{1}^{\prime },x_{2}^{\prime },\ldots ,x_{n}^{\prime }\right)\;\right]\;,$$

whose matrix elements are the above correlation functions. For the correlation operator, it is straightforward to define the order indices

(48) $$\omega \left({\hat{C}}_{n}\right)=\frac{\log||\;{\hat{C}}_{n}\;\left|\right|}{\log|\;\mathrm{Tr}{\hat{C}}_{n}\;|}$$

and the entanglement-production measure

(49) $$\epsilon ({\hat{C}}_{n})=\log\;\frac{||\;{\hat{C}}_{n}\;\left|\right|}{||\;{\hat{C}}_{n}^{⊗}\;\left|\right|}\;.$$

By choosing appropriate correlation functions one can quantify the properties of arbitrary physical systems.

## 7. Examples of Order Indices

Order indices are defined by the structure of the system state and can essentially vary under phase transformations [50]. Some examples of order indices that have been considered in literature are mentioned below.

### 7.1. Superconducting State

The structure of reduced density matrices have been analyzed in several works [6,8,9,47,49]. In the thermodynamic limit, the order indices of density matrices in a three-dimensional system are zero for the normal state and take the values

(50) $$\omega \left({\hat{\rho }}_{2n}\right)=\frac{1}{2}\;,\;\omega \left({\hat{\rho }}_{2n+1}\right)=\frac{n}{2n+1}\;,$$

for superconducting state. This corresponds to even long-range order.

### 7.2. Bose-Condensed System

Under the usual Bose–Einstein condensation into the state with zero momentum, in the thermodynamic limit in three dimensions, all order indices grow from zero to one, becoming

(51) $$\omega ({\hat{\rho }}_{n})=1\;.$$

This corresponds to the total long-range order [47,49]. Developing long-range order strongly influences the structure of correlation functions and density matrices [51,52,53,54].

### 7.3. Even Bose Condensate

There are theoretical speculations [55,56,57,58,59,60,61,62,63] that in Bose systems there can develop the so-called even condensate, with the formation of pairs similar to those for Fermi systems in the superconducting state, when

(52) $$\omega \left({\hat{\rho }}_{2n}\right)=\frac{1}{2}\;,$$

thus exhibiting even long-range order.

### 7.4. Finite-Momentum Condensate

If the condensed state is characterized not by zero momentum but by a momentum with finite absolute value and random direction [51,64,65,66], then the system possesses mid-range order with the order indices that, depending on dimensionality *d*, are

(53) $$\omega \left({\hat{\rho }}_{n}\right)=\frac{1}{d}\;.$$

For d>1 this implies a mid-range order [10].

### 7.5. Two-Dimensional Systems

Two-dimensional systems below the Berezinskii–Kosterlitz–Thouless transition [4,5] temperature $T_{K}$ possess the order indices [6]

(54) $$\omega \left({\hat{\rho }}_{n}\right)=1\;-\;\frac{\eta }{4}\;\left(d=2,\;T<T_{K}\right)\;,$$

where η is the exponent describing the behavior of the pair correlation function at large distance. In that case

$$\frac{1}{4}\leq \eta \leq \frac{1}{3}\;,$$

so that there exists a mid-range order with the order indices

$$\frac{11}{12}\leq \omega \left({\hat{\rho }}_{n}\right)\leq \frac{15}{16}\;\left(d=2,\;T<T_{K}\right)\;.$$

Mid-range order also develops in two-dimensional antiferromagnets in a strong magnetic field [67,68].

### 7.6. Critical Point

At the point of a second-order phase transition, we have [6]

(55) $$\omega \left({\hat{\rho }}_{n}\right)=\frac{d+2-\eta }{2d}\;\left(T=T_{c}\right)\;,$$

with η being the exponent characterizing the pair correlation function. For different dimensions, this gives

(56) $$\omega \left({\hat{\rho }}_{n}\right)=\frac{4-\eta }{4}\;\left(d=2,\;T=T_{c}\right)\;,\;\omega \left({\hat{\rho }}_{n}\right)=\frac{5-\eta }{6}\;\left(d=3,\;T=T_{c}\right)\;,$$

which implies mid-range order. In particular, for the two-dimensional Ising model, we have [69,70,71] η=1/4, hence

(57) $$\omega \left({\hat{\rho }}_{n}\right)=\frac{15}{16}\;\left(d=2,\;\mathrm{Ising},\;T=T_{c}\right)\;.$$

For the three-dimensional Heisenberg model, η=0.036, therefore

(58) $$\omega \left({\hat{\rho }}_{n}\right)=0.827\;\left(d=3,\;\mathrm{Heisenberg},\;T=T_{c}\right)\;.$$

### 7.7. Tonks–Girardeau Gas

One-dimensional systems usually do not exhibit long-range order, although they can possess mid-range order. As an example, let us consider the Tonks–Girardeau gas [72]. This is a one-dimensional system of impenetrable bosons described by the Hamiltonian

(59) $$\hat{H}=-\;\frac{1}{2m}\sum_{i=1}^{N}\frac{\partial^{2}}{\partial x_{i}^{2}}\;+\;\sum_{i=1}^{N}U\left(x_{i}\right)\;,$$

where U(x) is an external potential, and complimented by the condition on the wave function

(60) $$\psi \left(x_{1},x_{2},\ldots ,x_{N},t\right)=0\;(|\;x_{i}-x_{j}\;\left|\leq a_{0}\right)\;,$$

where $a_{0}$ is an effective diameter of particles. Studying the properties of reduced density matrices [73,74,75,76] one concludes that the order indices are

(61) $$\omega \left({\hat{\rho }}_{n}\right)=\frac{1}{2}$$

for both homogeneous as well as for systems trapped in power-law potentials. This shows that the Tonks–Girardeau gas possesses a mid-range order.

### 7.8. Lieb–Liniger Model

This is a one-dimensional model described by the Hamiltonian [77]

(62) $$\hat{H}=-\;\frac{1}{2m}\sum_{i=1}^{N}\frac{\partial^{2}}{\partial x_{i}^{2}}+\Phi_{0}\sum_{i\neq j}\delta \left(x_{i}-x_{j}\right),$$

with point-like interactions of finite strength $\Phi_{0}$. The characteristic dimensionless interaction parameter is defined by the ratio of the typical potential energy to the typical kinetic energy

$$E_{K}=\frac{\rho^{2}}{2m}\;\left(\rho \equiv \frac{N}{L}\right)\;,$$

which gives

(63) $$\gamma \equiv \frac{\rho \Phi_{0}}{E_{K}}=\frac{2m}{\rho }\;\Phi_{0}\;.$$

The model is exactly solvable by Bethe ansatz [77,78], which provides the exact expression of the many-body eigenfunctions [79]. At zero temperature, the ground-state energy, the sound velocity *c*, and other equilibrium quantities can be expressed in terms of the solution of the Lieb–Liniger integral equations [77]. The other useful quantity is the Luttinger parameter [80]

(64) $$M\equiv \frac{v_{F}}{c}\;\left(v_{F}\equiv \frac{\pi \rho }{m}\right)\;,$$

in which $v_{F}$ is the Fermi velocity and *c* is the sound velocity. Note that this parameter reminds the effective Mach number used for characterizing statistical systems [81]. Solving the Lieb–Liniger integral equations one has access to the sound velocity *c* and the Luttinger parameter *M* for any values of the coupling constant [82,83,84,85].

The order index of the first-order density matrix can be estimated [86] as

(65) $$\omega \left({\hat{\rho }}_{1}\right)=1\;-\;\frac{1}{2M}\;.$$

For small γ, one has

$$c≃\frac{\rho }{m}\;\sqrt{\gamma }\;,\;M≃\frac{\pi }{\sqrt{\gamma }}\;\left(\gamma \to 0\right)\;,$$

so that

(66) $$\omega \left({\hat{\rho }}_{1}\right)≃1\;-\;\frac{\sqrt{\gamma }}{2\pi }\;\left(\gamma \to 0\right)\;.$$

While for strong interactions, when

$$c≃v_{F}\;,\;M≃1\;\left(\gamma \to \infty \right)\;,$$

the order index tends to that of the Tonks–Girardeau gas,

(67) $$\omega \left({\hat{\rho }}_{1}\right)≃\frac{1}{2}\;\left(\gamma \to \infty \right)\;.$$

As we see, the Lieb–Liniger model demonstrates a mid-range order. Formally, the order index tends to one in the limit of the ideal gas, when γ=0. However, the ideal one-dimensional uniform gas is unstable [87,88].

### 7.9. Finite Systems

Strictly speaking, usually long-range order can arise only in thermodynamic limit. In finite systems, order parameters not always can be rigorously defined. However, the order indices can be well defined for any finite system. It is reasonable to expect that in finite systems, instead of long-range order, there could exist only mid-range or short-range order. In that case, the order indices vary in the interval

(68) $$0\leq \omega \left({\hat{\rho }}_{n}\right)<1\;\left(\mathrm{Bose}\right)\;,\;0\leq \omega \left({\hat{\rho }}_{n}\right)<\frac{1}{2}\;\left(\mathrm{Fermi}\right)\;,$$

depending on the number of particles in the system, on the interaction strength, and on other system parameters [89,90]. We shall discuss the details of calculating the order indices for systems with a finite, although large, number of particles in Section 9 and Section 10.

## 8. Examples of Entanglement Production

Now we shall give several examples of the entanglement-production measure calculated [39,91] according to Section 4. For bipartite states, it is also possible to find the entanglement entropy

(69) $$S\left({\hat{\rho }}_{i}\right)=-{\mathrm{Tr}}_{H_{i}}{\hat{\rho }}_{i}\ln{\hat{\rho }}_{i}\;,$$

where

$${\hat{\rho }}_{i}={\mathrm{Tr}}_{H/H_{i}}\hat{\rho }$$

is a partially traced statistical operator. The latter can be compared with the entanglement-production measure in Equation (23). In general, measures in Equations (23) and (69) do not need to be equal. In addition, the entanglement entropy in Equation (69) can be defined only for bipartite states, while the entanglement-production measure in Equation (23) can be defined for arbitrary states and operators.

### 8.1. Einstein–Podolsky–Rosen-States

The statistical operator of this entangled pure state is

$${\hat{\rho }}_{EPR}=|\;EPR\;\rangle \langle \;EPR\;|\;,$$

in which

$$|\;EPR\;\rangle =\frac{1}{\sqrt{2}}\;(\;|\;12\;\rangle \;\pm \;|\;21\;\rangle \;)\;.$$

This is a two-particle two-mode state. For the entanglement-production measure, we have

(70) $$\epsilon ({\hat{\rho }}_{EPR})=\log2\;.$$

This coincides with the entanglement entropy in Equation (69).

### 8.2. Bell States

This is also an entangled two-particle two-mode pure state with the statistical operator

$${\hat{\rho }}_{B}=|B\rangle \langle B|\;,$$

where

$$|B\rangle =\frac{1}{\sqrt{2}}\;(\;|\;11\;\rangle \;\pm \;|\;22\;\rangle \;)\;.$$

The measure in Equation (23)

(71) $$\epsilon ({\hat{\rho }}_{B})=\log2$$

equals the entanglement entropy in Equation (69).

### 8.3. Greenberger–Horne–Zeilinger States

This is an *N*-particle two-mode state

$${\hat{\rho }}_{GHZ}=|\;GHZ\;\rangle \langle \;GHZ\;|\;,$$

with

$$|\;GHZ\;\rangle =\frac{1}{\sqrt{2}}\;(\;|\;11\ldots 1\;\rangle \;\pm \;|\;22\ldots 2\;\rangle \;)\;.$$

For the measure in Equation (23), we get

(72) $$\epsilon ({\hat{\rho }}_{GHZ})=\left(N-1\right)\log2\;.$$

For the number of particles more than two, the entanglement entropy is not defined.

### 8.4. Multicat States

The statistical operator for the two-mode *N*-particle state

$${\hat{\rho }}_{MC}=\left|\;MC\;\rangle \langle \;MC\;\right|$$

is expressed through the multicat function

$$|\;MC\;\rangle =c_{1}|\;11\ldots 1\;\rangle \;+\;c_{2}|\;22\ldots 2\;\rangle \;,$$

where

$$|c_{1}{|}^{2}+{\left|c_{2}\right|}^{2}=1\;.$$

The entanglement-production measure becomes

(73) $$\epsilon ({\hat{\rho }}_{MC})=\left(1-N\right)\log\sup\{|c_{1}{|}^{2},\;|c_{2}{|}^{2}\left|\right\}\;.$$

Multicat states can be realized in multiparticle systems where each particle possesses two internal energy states, for instance, for trapped ions subject to the action of resonant laser beams [92,93] or for Bose-condensed neutral atoms under coherent Raman scattering [94]. Instead of internal single-particle states, one can use collective nonlinear states created by means of the resonant excitation of topological coherent modes in trapped Bose–Einstein condensates [95,96,97,98].

### 8.5. Multimode States

The state of *N* particles possessing $N_{M}$ modes each,

$${\hat{\rho }}_{MM}=|\;MM\;\rangle \langle \;MM\;|\;,$$

is formed by the multimode function

$$|\;MM\;\rangle =\sum_{n=1}^{N_{M}}c_{n}|nn\ldots n\rangle \;,$$

for which

$$\sum_{n=1}^{N_{M}}{\left|c_{n}\right|}^{2}=1\;.$$

This is a generalization of the multicat state to the case of *N* particles. The measure of entanglement production is

(74) $$\epsilon ({\hat{\rho }}_{MM})=\left(1-N\right)\log\underset{n}{\sup}{\left|c_{n}\right|}^{2}\;.$$

The multimode functions can be represented by coherent states [99].

### 8.6. Hatree–Fock States

The state with the statistical operator

$${\hat{\rho }}_{HF}=|\;HF\;\rangle \langle \;HF\;|\;$$

is formed by *N*-particle symmetrized or antisymmetrized functions

$$|\;HF\;\rangle =\frac{1}{\sqrt{N!}}\sum_{sym}|12\ldots N\rangle \;.$$

Such functions represent *N* identical particles, bosons or fermions, in *N* different energy states. The entanglement-production measure reads as

(75) $$\epsilon ({\hat{\rho }}_{HF})=\log\;\frac{N^{N}}{N!}\;.$$

In the case of two particles, the measure reduces to the that for the Einstein–Podolsky–Rosen state,

(76) $$\epsilon ({\hat{\rho }}_{HF})=\log2\;\left(N=2\right)\;.$$

For macroscopic systems, it tends to the expression

(77) $$\epsilon ({\hat{\rho }}_{HF})≃N\log e\;\left(N\gg 1\right)\;.$$

### 8.7. Reduced Statistical Operators

In the above examples, pure states are considered. The measure of entanglement production, being general, can be found for mixed states as well. To this end, we can study reduced statistical operators defined through partial traces of the Hartree–Fock state

$${\hat{\rho }}_{n}\equiv {\mathrm{Tr}}_{H_{n+1}}{\mathrm{Tr}}_{H_{n+2}}\ldots {\mathrm{Tr}}_{H_{N}}\;{\hat{\rho }}_{HF}\;,$$

with n=1,2,…,N−1. The entanglement-production measure for this operator is

(78) $$\epsilon ({\hat{\rho }}_{n})=\log\;\frac{\left(N-n\right)!N^{n}}{N!}\;.$$

For a large number of particles, this yields

(79) $$\epsilon ({\hat{\rho }}_{n})≃\frac{n(n-1)}{2N}\;\log e\;\left(N\gg 1\right)\;.$$

### 8.8. Gibbs States

An equilibrium system of *N* particles, characterized by a Hamiltonian *H* acting on a Hilbert space

$$H=\overset{N}{\underset{i=1}{⨂}}H_{i}\;,$$

is described by the Gibbs statistical operator

$$\hat{\rho }=\frac{1}{Z}\;e^{-\beta H}\;\left(Z\equiv {\mathrm{Tr}}_{H}e^{-\beta H}\right)\;,$$

in which β≡1/T is inverse temperature. The entanglement-production measure can be calculated as is explained in Section 4. For two-particle registers, represented by Ising and Heisenberg Hamiltonians, this was done in Refs. [39,91].

## 9. Werner Operator

The case of the Werner operator [100] is interesting since, depending on parametrization, it can represent a separable or entangled state. The operator has the form

(80) $${\hat{\rho }}_{W}=\frac{1}{(d^{2}-1)d}\;\left[\;\left(d-\gamma \right)\hat{1}+\left(\gamma d-1\right)\hat{\sigma }\;\right]\;,$$

with the unity operator

$$\hat{1}=\sum_{mn}\left|\;mn\;\rangle \langle \;nm\;\right|={\hat{1}}_{A}⨂{\hat{1}}_{B}\;,$$

where

$${\hat{1}}_{A}=\sum_{m}|\;m\;\rangle \langle \;m\;|\;,\;{\hat{1}}_{B}=\sum_{n}|\;n\;\rangle \langle \;n\;|\;,$$

and the flip operator

$$\hat{\sigma }=\sum_{mn}\left|\;mn\;\rangle \langle \;mn\;\right|=\sum_{mn}|\;m\;\rangle \langle \;n\;|⨂|\;n\;\rangle \langle \;m\;|\;.$$

The name of the latter comes from the property of the flip operator to flip functions:

$$\hat{\sigma }\;|\;mn\;\rangle =|\;nm\;\rangle \;.$$

The parametrization of the Werner operator is done by the parameter γ defined by the equation

$$\gamma ={\mathrm{Tr}}_{H}{\hat{\rho }}_{W}\hat{\sigma }\;\left({\mathrm{Tr}}_{H}{\hat{\rho }}_{W}=1\right)\;.$$

The partially traced operators are

$${\hat{\rho }}_{1}={\mathrm{Tr}}_{H_{2}}{\hat{\rho }}_{W}=\frac{1}{d}\;{\hat{1}}_{A}\;,\;{\hat{\rho }}_{2}={\mathrm{Tr}}_{H_{1}}{\hat{\rho }}_{W}=\frac{1}{d}\;{\hat{1}}_{B}\;.$$

These reduced operators are normalized to one,

$${\mathrm{Tr}}_{H_{1}}{\hat{\rho }}_{1}={\mathrm{Tr}}_{H_{2}}{\hat{\rho }}_{2}=1\;.$$

The nonentangling counterpart of the Werner operator is

$${\hat{\rho }}_{W}^{⊗}={\hat{\rho }}_{1}⨂{\hat{\rho }}_{2}=\frac{1}{d^{2}}\;\hat{1}\;,$$

which gives its norm

$$\left|\right|{\hat{\rho }}_{W}^{⊗}\left|\right|=\frac{1}{d^{2}}\;.$$

In view of the matrix elements

$$\langle \;mn\;|\hat{1}\;|\;nm\;\rangle =1\;,\;\langle \;mn\;|\hat{\sigma }\;|\;nm\;\rangle =\delta_{mn}\;,$$

we have

$$\langle \;mn\;|{\hat{\rho }}_{W}\;|\;nm\;\rangle =\frac{d-\gamma +\left(\gamma d-1\right)\delta_{mn}}{(d^{2}-1)d}\;.$$

Thence the norm of the Werner operator is

$$\left|\right|{\hat{\rho }}_{W}\left|\right|=\frac{1}{(d^{2}-1)d}\sup\left\{d-\gamma ,\;\left(1+\gamma \right)\left(d-1\right)\right\}\;.$$

Therefore for the entanglement-production measure we find

(81) $$\epsilon ({\hat{\rho }}_{W})=\log\left[\;\frac{d}{d^{2}-1}\sup\left\{d-\gamma ,\;\left(1+\gamma \right)\left(d-1\right)\right\}\;\right]\;.$$

The measure is positive for all values of γ, which means that the Werner operator is always entangling. At the same time, the positive partial transpose criterion [101,102] tells us that the Werner state is separable if and only if γ≥0, while it is entangled for γ<0. However, even being separable, the Werner operator is entangling, that is, producing entanglement.

The Werner operator is an explicit example of an operator that can be separable, although being entangling, as is discussed in Section 4. The entanglement entropy for the Werner operator is

$$S_{j}=-{\mathrm{Tr}}_{H_{j}}{\hat{\rho }}_{j}\ln{\hat{\rho }}_{j}=\frac{1}{d}\;\ln d\;.$$

## 10. Bose–Einstein Condensation

The aim of the present section is twofold. First, it shows explicitly how the order indices and the entanglement-production measure can be calculated for a system with a phase transformation for a large, however finite, number of particles. Second, it is demonstrated that both these characteristics, order indices as well as entanglement production, simultaneously change under phase transitions.

The correct description of a Bose-condensed system requires the validity of the following important stipulations. (i) The theory has to respect conservation laws and thermodynamic relations. (ii) The excitation spectrum must be gapless. (iii) The Bose–Einstein condensation has to be a phase transition of second order. (iv) The system must be stable, satisfying the necessary stability conditions. (v) Reasonable quantitative agreement with experiments is required. The validity of these stipulations can be achieved in the self-consistent approach based on representative statistical ensembles [103,104,105,106] which we use here.

In the presence of Bose–Einstein condensate, the boson field operators acquire the Bogolubov shift [107,108], being represented as the sum

(82) $$\hat{\psi }\left(r\right)=\eta \left(r\right)+\psi_{1}\left(r\right)\;,$$

in which η is the condensate wave function and $\psi_{1}$ is the field operator of uncondensed particles. The field operators are also functions of time, which are not shown explicitly for the sake of notation compactness. The functional variables η and ψ are orthogonal with each other,

(83) $$\int \eta^{*}\left(r\right)\psi_{1}\left(r\right)\;dr=0$$

and satisfy the conditions

(84) $$\eta \left(r\right)=\langle \;\hat{\psi }\left(r\right)\;\rangle \;,\;\langle \;{\hat{\psi }}_{1}\left(r\right)\;\rangle =0\;.$$

The condensate function is normalized to the number of condensed particles,

(85) $$N_{0}=\int {\left|\;\eta \left(r\right)\;\right|}^{2}\;dr\;,$$

while the number of uncondensed particles is

(86) $$N_{1}=\int \langle \;{\hat{\psi }}_{1}^{†}\left(r\right)\psi_{1}\left(r\right)\;\rangle \;dr\;.$$

The first-order density matrix reads as

(87) $$\rho \left(r,r^{\prime }\right)=\eta^{*}\left(r^{\prime }\right)\eta \left(r\right)+\langle \;{\hat{\psi }}_{1}^{†}\left(r^{\prime }\right)\psi_{1}\left(r\right)\;\rangle \;.$$

Its eigenvalues can be represented through the integral

(88) $$N_{k}=\int \varphi_{k}^{*}\left(r\right)\rho \left(r,r^{\prime }\right)\varphi_{k}\left(r^{\prime }\right)\;drdr^{\prime }\;,$$

in which $\varphi_{k}$ are the natural orbitals [47]. Taking account of Equation (87) leads to the sum

(89) $$N_{k}=N_{0k}+n_{k}\;,$$

where

$$N_{0k}\equiv {\left|\;\int \eta^{*}\left(r\right)\varphi_{k}\left(r\right)\;dr\;\right|}^{2}$$

and

$$n_{k}\equiv \langle \;a_{k}^{†}a_{k}\;\rangle \;,\;a_{k}\equiv \int \varphi_{k}^{*}\left(r\right)\psi_{1}\left(r\right)\;dr\;.$$

Thus we obtain the norm

(90) $$||\;{\hat{\rho }}_{1}\;\left|\right|=\underset{k}{\sup}\left(N_{0k}+n_{k}\right)\;.$$

In the absence of the condensate, η=0 and $N_{0k}=0$.

In the presence of the condensate, there also exists the so-called anomalous average

(91) $$\sigma_{1}\left(r_{1},r_{2}\right)\equiv \langle \;\psi_{1}\left(r_{2}\right)\psi_{1}\left(r_{1}\right)\;\rangle =\sigma_{1}\left(r_{2},r_{1}\right)\;,$$

with the property

$$\sigma_{1}^{*}\left(r_{1},r_{2}\right)\equiv \langle \;\psi_{1}^{†}\left(r_{1}\right)\psi_{1}^{†}\left(r_{2}\right)\;\rangle =\sigma_{1}^{*}\left(r_{2},r_{1}\right)\;.$$

If the system is uniform, then

(92) $$\eta \left(r\right)=\sqrt{\rho_{0}}\;\left(\rho_{0}\equiv \frac{N_{0}}{V}\right)$$

and it follows

(93) $$N_{0k}=N_{0}\delta_{k0}\;.$$

Considering the second-order density matrix, we shall use the simplified notation writing just *j* instead of $r_{j}$ and $j^{\prime }$ instead of $r_{j}^{\prime }$. For instance,

(94) $$\rho_{1}\left(1,2\right)\equiv \rho_{1}\left(r_{1},r_{2}\right)=\langle \;\psi_{1}^{†}\left(r_{2}\right)\psi_{1}\left(r_{1}\right)\;\rangle \;.$$

Then the second-order density matrix reads as

(95) $$\rho_{2}\left(1,2,1^{\prime },2^{\prime }\right)=\eta^{*}\left(2^{\prime }\right)\eta^{*}\left(1^{\prime }\right)\eta \left(1\right)\eta \left(2\right)+\rho_{1}\left(1,2^{\prime }\right)\eta^{*}\left(1^{\prime }\right)\eta \left(2\right)+\rho_{1}\left(1,1^{\prime }\right)\eta^{*}\left(2^{\prime }\right)\eta \left(2\right)+\;+\rho_{1}\left(2,2^{\prime }\right)\eta^{*}\left(1^{\prime }\right)\eta \left(1\right)+\rho_{1}\left(2,1^{\prime }\right)\eta^{*}\left(2^{\prime }\right)\eta \left(1\right)+\sigma_{1}\left(1,2\right)\eta^{*}\left(2^{\prime }\right)\eta^{*}\left(1^{\prime }\right)+\sigma_{1}^{*}\left(1^{\prime },2^{\prime }\right)\eta \left(1\right)\eta \left(2\right)+\;+\langle \;\psi_{1}^{†}\left(2^{\prime }\right)\psi_{1}^{†}\left(1^{\prime }\right)\psi_{1}\left(1\right)\;\rangle \;\eta \left(2\right)+\langle \;\psi_{1}^{†}\left(2^{\prime }\right)\psi_{1}^{†}\left(1^{\prime }\right)\psi_{1}\left(2\right)\;\rangle \eta \left(1\right)+\;+\langle \;\psi_{1}^{†}\left(2^{\prime }\right)\psi_{1}\left(1\right)\psi_{1}\left(2\right)\;\rangle \;\eta^{*}\left(1^{\prime }\right)+\langle \;\psi_{1}^{†}\left(1^{\prime }\right)\psi_{1}\left(1\right)\psi_{1}\left(2\right)\;\rangle \;\eta^{*}\left(2^{\prime }\right)+\langle \;\psi_{1}^{†}\left(2^{\prime }\right)\psi_{1}^{†}\left(1^{\prime }\right)\psi_{1}\left(1\right)\psi_{1}\left(2\right)\;\rangle \;.$$

In what follows, let us consider a uniform system, when Equation (92) holds true. In that case, the natural orbitals are plane waves. By employing the Hartree–Fock–Bogolubov decoupling reduces the second-order density matrix to the expression

(96) $$\rho_{2}\left(1,2,1^{\prime },2^{\prime }\right)=\rho_{0}^{2}+\rho_{0}\left[\;\rho_{1}\left(1,2^{\prime }\right)+\rho_{1}\left(1,1^{\prime }\right)+\rho_{1}\left(2,2^{\prime }\right)+\rho_{1}\left(2,1^{\prime }\right)+\sigma_{1}\left(1,2\right)+\sigma_{1}^{*}\left(1^{\prime },2^{\prime }\right)\;\right]+\;+\rho_{1}\left(1,2^{\prime }\right)\rho_{1}\left(2,1^{\prime }\right)+\rho_{1}\left(2,2^{\prime }\right)\rho_{1}\left(1,1^{\prime }\right)+\sigma_{1}^{*}\left(1^{\prime },2^{\prime }\right)\sigma_{1}\left(1,2\right)\;.$$

The matrix element in Equation (94) becomes

(97) $$\rho_{1}\left(r,r^{\prime }\right)=\frac{1}{V}\sum_{k\neq 0}n_{k}e^{ik\cdot (r-r^{\prime })}\;,$$

where

$$n_{k}\equiv \langle \;a_{k}^{†}a_{k}\;\rangle =\frac{1}{V}\int \rho_{1}\left(r^{\prime },r\right)e^{ik\cdot (r-r^{\prime })}\;drdr^{\prime }\;.$$

Similarly, the anomalous average in Equation (91) takes the form

(98) $$\sigma_{1}\left(r,r^{\prime }\right)=\frac{1}{V}\sum_{k\neq 0}\sigma_{k}e^{ik\cdot (r-r^{\prime })}\;,$$

with

$$\sigma_{k}\equiv \langle \;a_{k}a_{-k}\;\rangle =\frac{1}{V}\int \sigma_{1}\left(r^{\prime },r\right)e^{ik\cdot (r-r^{\prime })}\;drdr^{\prime }\;.$$

For the norm of the second-order density operator, we get

(99) $$||\;{\hat{\rho }}_{2}\;\left|\right|=\underset{kp}{\sup}\left(N_{0}^{2}\delta_{k0}\delta_{p0}+2n_{k}n_{p}+\sigma_{k}\sigma_{p}\right)\;.$$

In that way, we find the order indices for the density operators of first order,

(100) $$\omega \left({\hat{\rho }}_{1}\right)=\frac{\log||\;{\hat{\rho }}_{1}\;\left|\right|}{\log|\;\mathrm{Tr}{\hat{\rho }}_{1}\;|}=\frac{\log\sup_{k}N_{k}}{\log N}\;,$$

in which

$$N_{k}=N_{0}\delta_{k0}+n_{k}\;,$$

and of second order

(101) $$\omega \left({\hat{\rho }}_{2}\right)=\frac{\log||\;{\hat{\rho }}_{2}\;\left|\right|}{\log|\;\mathrm{Tr}{\hat{\rho }}_{2}\;|}=\frac{\log\sup_{kp}N_{kp}}{2\log N}\;,$$

where

$$N_{kp}=N_{0}^{2}\delta_{k0}\delta_{p0}+2n_{k}n_{p}+\sigma_{k}\sigma_{p}\;.$$

For the entanglement-production measure, we obtain

(102) $$\epsilon ({\hat{\rho }}_{2})=\log\;\frac{||\;{\hat{\rho }}_{2}\;\left|\right|}{||\;{\hat{\rho }}_{2}^{⊗}\;\left|\right|}=\log\;\frac{\sup_{kp}N_{kp}}{{\left(\sup_{k}N_{k}\right)}^{2}}\;.$$

These quantities can be calculated [89,90] as functions of the number of particles and other system parameters.

For illustration, let us consider the limiting case of N→∞. Then, for temperatures below the condensation temperature $T_{c}$, we find that

$$\underset{k}{\sup}N_{k}≃N_{0}\propto N\;,\;\underset{kp}{\sup}N_{kp}≃N_{0}^{2}\propto N^{2}\;\left(T<T_{c}\right)\;,$$

while $\sup_{k}n_{k}\propto N^{1/3}$. At temperatures above the condensation temperature $T_{c}$, we have

$$\underset{k}{\sup}N_{k}=\underset{k}{\sup}n_{k}\;,\;\underset{kp}{\sup}N_{kp}=2\underset{k}{\sup}n_{k}^{2}\;\left(T>T_{c}\right)\;.$$

Therefore the studied order indices are

(103) $$\begin{array}{c} \omega \left({\hat{\rho }}_{1}\right)=\omega \left({\hat{\rho }}_{2}\right)=\left\{\begin{array}{cc} 1,\; & \;T<T_{c}\\ 0,\; & \;T>T_{c} \end{array}\right. \end{array}$$

and the entanglement-production measure is

(104) $$\epsilon ({\hat{\rho }}_{2})=\left\{\begin{array}{cc} 0,\; & \;T<T_{c}\\ \log2,\; & \;T>T_{c} \end{array}\;.\right.$$

At the phase transition point, both these quantities experience noticeable change. The appearance of order is accompanied by the reduction of the entanglement production.

## 11. Magnetic Transitions

Magnetic systems are characterized by spin operators $S_{j}$ located at lattice sites enumerated by j=1,2,…,N. Instead of density matrices defined through field operators, we need now to introduce correlation matrices, as in Section 6, composed of spin operators [10]. Thus the first-order spin correlation matrix

(105) $${\hat{C}}_{1}=\left[\;C_{ij}\;\right]$$

is composed of the matrix elements

(106) $$C_{ij}=\langle \;S_{i}\cdot S_{j}\;\rangle \;.$$

The second-order spin correlation matrix

(107) $${\hat{C}}_{2}=\left[\;C_{ijmn}\;\right]$$

is formed by the correlation functions

(108) $$C_{ijmn}=\langle \;S_{i}\left(S_{j}\cdot S_{m}\right)S_{n}\;\rangle \;.$$

Keeping in mind that

$$S_{j}^{2}=S\left(S+1\right)\;,$$

we have the traces

(109) $$\mathrm{Tr}{\hat{C}}_{1}=\sum_{j}C_{jj}=S\left(S+1\right)N$$

and

(110) $$\mathrm{Tr}{\hat{C}}_{2}=\sum_{ij}C_{ijji}={\left[\;S\left(S+1\right)N\;\right]}^{2}\;.$$

In calculating the matrix norms, we use the natural lattice orbital

(111) $$\varphi_{k}\left(a_{j}\right)=\frac{1}{\sqrt{N}}\;e^{ik\cdot a_{j}}\;.$$

Then the norm of the first-order correlation matrix is

(112) $$||\;{\hat{C}}_{1}\;\left|\right|=\underset{k}{\sup}\left|\;\sum_{ij}\varphi_{k}^{*}\left(a_{i}\right)C_{ij}\varphi_{k}\left(a_{j}\right)\;\right|\;.$$

Employing the properties

$$C_{ijjn}=S\left(S+1\right)C_{in}\;,\;{\left|\;\varphi_{k}\left(a_{j}\right)\;\right|}^{2}=\frac{1}{N}\;,$$

and separating the terms with coinciding and non-coinciding lattice sites, we get

(113) $$||\;{\hat{C}}_{1}\;\left|\right|=\underset{k}{\sup}\left|\;S\left(S+1\right)+\sum_{i\neq j}\varphi_{k}^{*}\left(a_{i}\right)C_{ij}\varphi_{k}\left(a_{j}\right)\;\right|\;.$$

Similarly, for the second-order spin correlation matrix, we have the norm

(114) $$||\;{\hat{C}}_{2}\;\left|\right|=\underset{kp}{\sup}\left|\;\sum_{ijmn}\varphi_{k}^{*}\left(a_{i}\right)\varphi_{p}^{*}\left(a_{j}\right)C_{ijmn}\varphi_{p}\left(a_{m}\right)\varphi_{k}\left(a_{n}\right)\;\right|\;,$$

which leads to

(115) $$||\;{\hat{C}}_{2}\;\left|\right|=\underset{kp}{\sup}\left|\;{\left[\;S\left(S+1\right)\;\right]}^{2}+2S\left(S+1\right)\sum_{i\neq j}\varphi_{k}^{*}\left(a_{i}\right)C_{ij}\varphi_{k}\left(a_{j}\right)+\right.\;+\left.\sum_{i\neq n}\;\sum_{j\neq m}\varphi_{k}^{*}\left(a_{i}\right)\varphi_{p}^{*}\left(a_{j}\right)C_{ijmn}\varphi_{p}\left(a_{m}\right)\varphi_{k}\left(a_{n}\right)\;\right|\;.$$

Defining the magnetization

(116) $$M=\langle \;S_{j}\;\rangle \;,$$

for different lattice sites, one can resort to the mean-field approximation

(117) $$\langle \;S_{i}\cdot S_{j}\;\rangle =\langle \;S_{i}\;\rangle \langle \;S_{j}\;\rangle =M^{2}\;\left(i\neq j\right)\;.$$

Then we have

$$\begin{array}{c} C_{ij}=\left\{\begin{array}{cc} M^{2},\; & \;i\neq j\\ S(S+1),\; & \;i=j\;, \end{array}\right. \end{array}$$

which gives

(118) $$||\;{\hat{C}}_{1}\;\left|\right|=S\left(S+1\right)+\left(N-1\right)M^{2}\;.$$

Depending on the number of coinciding and non-coinciding lattice sites, we find

$$\langle \;S_{i}\left(S_{j}\cdot S_{m}\right)S_{n}\;\rangle =\langle \;S_{i}\cdot S_{n}\;\rangle \langle \;S_{j}\cdot S_{m}\;\rangle \;\left(i\neq n,\;j\neq m,\;j\neq n,\;m\neq n\right)\;,\;\langle \;S_{j}\left(S_{j}\cdot S_{m}\right)S_{n}\;\rangle =\langle \;S_{j}\cdot S_{j}\;\rangle \langle \;S_{m}\cdot S_{n}\;\rangle \;\left(j\neq n,\;j\neq m,\;m\neq n\right)\;,\;\langle \;S_{i}\left(S_{j}\cdot S_{n}\right)S_{n}\;\rangle =\langle \;S_{i}\cdot S_{j}\;\rangle \langle \;S_{n}\cdot S_{n}\;\rangle \;\left(i\neq n,\;j\neq n\right)\;,\;\langle \;S_{j}\left(S_{j}\cdot S_{n}\right)S_{n}\;\rangle =\langle \;S_{j}\cdot S_{j}\;\rangle \langle \;S_{n}\cdot S_{n}\;\rangle \;\left(j\neq n\right)\;.$$

This leads to the matrix elements

$$C_{ijmn}=M^{4}\;\left(i\neq n,\;j\neq m,\;j\neq n,\;m\neq n\right)\;,\;C_{jjmn}=S\left(S+1\right)M^{2}\;\left(j\neq n,\;j\neq m,\;m\neq n\right)\;,\;C_{ijnn}=S\left(S+1\right)M^{2}\;\left(i\neq n,\;j\neq n\right)\;,\;C_{jjnn}={\left[\;S\left(S+1\right)\;\right]}^{2}\;\left(j\neq n\right)\;.$$

Therefore we obtain

(119) $$||\;{\hat{C}}_{2}\;\left|\right|=2{\left[\;S\left(S+1\right)\;\right]}^{2}+4\left(N-1\right)S\left(S+1\right)M^{2}+{\left(N-1\right)}^{2}M^{4}\;.$$

The nonentangling counterpart of the second-order correlation matrix,

(120) $${\hat{C}}_{2}^{⊗}={\hat{C}}_{1}⨂{\hat{C}}_{2}\;,$$

for which

(121) $$\mathrm{Tr}{\hat{C}}_{2}^{⊗}=\mathrm{Tr}{\hat{C}}_{2}={\left[\;S\left(S+1\right)N\;\right]}^{2},$$

results in the norm

(122) $$||\;{\hat{C}}_{2}^{⊗}\;||=||\;{\hat{C}}_{1}\;{\left|\right|}^{2}={\left[\;S\left(S+1\right)\;\right]}^{2}+2\left(N-1\right)S\left(S+1\right)M^{2}+{\left(N-1\right)}^{2}M^{4}\;.$$

The norms are essentially different for the magnetically ordered state, when a nonzero magnetization is present, and for the paramagnetic state without average magnetization:

$$\begin{array}{c} ||\;{\hat{C}}_{1}\;\left|\right|=\left\{\begin{array}{cc} NM^{2},\; & \;|M|>0\\ S(S+1), & \;M=0 \end{array}\;,\right. \end{array}$$

$$\begin{array}{c} ||\;{\hat{C}}_{2}\;\left|\right|=\left\{\begin{array}{cc} N^{2}M^{4},\; & \;|M|>0\\ 2{\left[\;S\left(S+1\right)\;\right]}^{2},\; & \;M=0 \end{array}\;,\right. \end{array}$$

(123) $$\begin{array}{c} ||\;{\hat{C}}_{2}^{⊗}\;\left|\right|=\left\{\begin{array}{cc} N^{2}M^{4},\; & \;|M|>0\\ \;{\left[\;S\left(S+1\right)\;\right]}^{2},\; & \;M=0 \end{array}\;,\right. \end{array}$$

For the order indices

(124) $$\omega \left({\hat{C}}_{n}\right)=\frac{\log|\;{\hat{C}}_{n}\;|}{\log|\;\mathrm{Tr}{\hat{C}}_{n}\;|}\;,$$

we have

(125) $$\begin{array}{c} \omega \left({\hat{C}}_{1}\right)=\omega \left({\hat{C}}_{2}\right)=\left\{\begin{array}{cc} 1,\; & \;|M|>0\\ 0, & \;M=0 \end{array}\;.\right. \end{array}$$

For the entanglement-production measure

(126) $$\epsilon ({\hat{C}}_{2})=\log\;\frac{||\;{\hat{C}}_{2}\;\left|\right|}{||\;{\hat{C}}_{2}^{⊗}\;\left|\right|}\;,$$

we find

(127) $$\begin{array}{c} \epsilon ({\hat{C}}_{2})=\left\{\begin{array}{cc} 0,\; & \;|M|>0\\ \log2, & \;M=0 \end{array}\;.\right. \end{array}$$

Here a large system, with N≫1 is assumed.

Again we see that the entanglement production diminishes upon arising order and increases for a disordered state.

## 12. Diagonal Order

In the previous Section 10 and Section 11, the cases of phase transformations with the arising off-diagonal order are analyzed. There exists an opinion that the transitions with the arising diagonal order have to be treated differently, since the related reduced density matrices behave in a different way. However, in the approach based on the order indices, there is no difference in the method of treating any type of phase transition. What needed is to define the appropriate correlation matrix [10]. In the present section, we illustrate this for the solid-liquid transition that is the most known transition exhibiting diagonal order.

Under the solidification–melting phase transition, what changes is the particle density

(128) $$\rho \left(r\right)=\langle \;\hat{\rho }\left(r\right)\;\rangle$$

that is the statistical average of the density operator

(129) $$\hat{\rho }\left(r\right)\equiv \psi^{†}\left(r\right)\psi \left(r\right)\;.$$

In the liquid state, the particle density is constant in space, being equal to the average density

(130) $$\rho =\frac{1}{V}\int \rho \left(r\right)\;dr=\frac{N}{V}\;.$$

In the solid state, the density is nonuniform, having minima and maxima.

We can label the points of the density maxima by $a_{j}$, enumerating them by the index $j=1,2,\ldots ,N_{L}$, thus defining them by the condition

(131) $$\max_{r}\rho \left(r\right)=\rho \left(a_{j}\right)\;.$$

Generally, the points of maxima do not need to form a periodic structure, but can be randomly located, as in amorphous solids. For crystalline structures, the points of density maxima form a periodic crystalline lattice, where the set of $\left\{a_{j}\right\}$ fixes lattice sites. Below, we shall consider a crystalline lattice, where the points of density maxima are identical to each other, so that

(132) $$\rho \left(a_{j}\right)=\rho \left(a\right)\;,$$

with a being any site from the set of lattice sites. This is not principal for the approach, but just simplifies some notations.

Let us introduce the operator

(133) $$\hat{A}\left(a_{j}\right)\equiv \hat{\rho }\left(a_{j}\right)-\rho \;.$$

Its average is

$$\langle \;\hat{A}\left(a_{j}\right)\;\rangle =\rho \left(a\right)-\rho \;.$$

With these operators, it is straightforward to define the correlation functions

(134) $$D_{ij}=\langle \;\hat{A}\left(a_{i}\right)\hat{A}\left(a_{j}\right)\;\rangle \;.$$

These functions play the role of matrix elements of the correlation matrix

(135) $${\hat{D}}_{1}=\left[\;D_{ij}\;\right]\;,$$

for which

(136) $$D_{ij}=\langle \;\hat{\rho }\left(a_{i}\right)\hat{\rho }\left(a_{j}\right)\;\rangle -2\rho \rho \left(a\right)+\rho^{2}\;.$$

The trace of this matrix is

(137) $$\mathrm{Tr}{\hat{D}}_{1}=\sum_{j}D_{jj}=N_{L}\left[\langle \;\hat{\rho }{\left(a\right)}^{2}\;\rangle -2\rho \rho \left(a\right)+\rho^{2}\right]\;,$$

with $N_{L}$ being the number of lattice sites. This trace can be rewritten as

(138) $$\mathrm{Tr}{\hat{D}}_{1}=N_{L}\left\{{\left[\;\rho \left(a\right)-\rho \;\right]}^{2}+\mathrm{var}\hat{\rho }\left(a\right)\;\right\}\;,$$

where

$$\mathrm{var}\hat{\rho }\left(a\right)\equiv \langle \;\hat{\rho }{\left(a\right)}^{2}\;\rangle -\rho {\left(a\right)}^{2}$$

is the density variance at a lattice site. For different sites, one can use the Hartree decoupling

$$\langle \;\hat{\rho }\left(a_{i}\right)\hat{\rho }\left(a_{j}\right)\;\rangle =\langle \;\hat{\rho }\left(a_{i}\right)\;\rangle \langle \;\hat{\rho }\left(a_{j}\right)\;\rangle \;\left(i\neq j\right)$$

that is known to provide a good description for crystals [109,110,111,112,113,114]. Keeping in mind the lattice function in Equation (111), for the norm of the matrix, we find

(139) $$||\;{\hat{D}}_{1}\;\left|\right|=\rho {\left(a\right)}^{2}+\mathrm{var}\hat{\rho }\left(a\right)+N_{L}{\left[\;\rho \left(a\right)-\rho \;\right]}^{2}\;.$$

In this way, we come to the order index

(140) $$\omega \left({\hat{D}}_{1}\right)=\frac{\log||\;{\hat{D}}_{1}\;\left|\right|}{\log|\;\mathrm{Tr}{\hat{D}}_{1}\;|}=\;=\frac{\log\{N_{L}{\left[\rho \left(a\right)-\rho \right]}^{2}+\rho {\left(a\right)}^{2}+\mathrm{var}\hat{\rho }\left(a\right)\}}{\log N_{L}+\log\left\{{\left[\rho \left(a\right)-\rho \right]}^{2}+\mathrm{var}\hat{\rho }\left(a\right)\right\}}\;.$$

For a solid, this reduces to

(141) $$\omega \left({\hat{D}}_{1}\right)=1+\frac{2\log[\rho (a)-\rho ]}{\log N_{L}}\;,\;\rho \left(a\right)>\rho \;,$$

while for a liquid, it gives

(142) $$\omega \left({\hat{D}}_{1}\right)=\frac{\log\{\rho {\left(a\right)}^{2}+\mathrm{var}\hat{\rho }\left(a\right)\}}{\log N_{L}}\;,\;\rho \left(a\right)=\rho \;.$$

These expressions can be used for both, finite systems as well as for macroscopic systems with a large number of particles. Note that finite crystalline structures can be stable, or metastable, even in low dimensions, forming crystalline chains and planes [115] for which the Lindemann stability criterion [116] is valid. In the thermodynamic limit, we obtain

(143) $$\begin{array}{c} \omega \left({\hat{D}}_{1}\right)=\left\{\begin{array}{cc} 1,\; & \;\mathrm{solid}\\ 0,\; & \;\mathrm{liquid} \end{array}\right.\;\left(N_{L}\to \infty \right)\;. \end{array}$$

Similarly, the characteristics of higher-order correlation matrices ${\hat{D}}_{n}$ can be calculated. The overall situation is analogous to the cases of phase transitions treated in Section 10 and Section 11.

## 13. Dynamical Effects

Order indices and entanglement production can vary with time. Time dependence can come through equations of motion. If the operator obeys a unitary evolution, the order indices do not change with time, although the entanglement production can vary since the non-entangling operator, generally, does not follow the unitary evolution. Reduced density matrices and correlation matrices, in general, also are not governed by unitary time dependence. Therefore the order indices and entanglement-production measure, say for reduced density matrices of nonequilibrium systems, do depend on time,

(144) $$\omega \left({\hat{\rho }}_{n}\left(t\right)\right)=\frac{\log||\;{\hat{\rho }}_{n}\left(t\right)\;\left|\right|}{\log|\;\mathrm{Tr}{\hat{\rho }}_{n}\left(t\right)\;|}\;,\;\epsilon ({\hat{\rho }}_{n}\left(t\right))=\log\;\frac{||\;{\hat{\rho }}_{n}\left(t\right)\;\left|\right|}{||\;{\hat{\rho }}_{n}^{⊗}\left(t\right)\;\left|\right|}\;.$$

### 13.1. Multitrap Multimode States

As an example, let us consider the case of multiple traps filled by Bose–Einstein condensate. This can be realized with an optical lattice having deep wells, where condensate clouds are located. Thus, cold rubidium ${}^{87}$Rb atoms were loaded [117,118] into an optical lattice, with adjacent sites spaced so that these sites were practically independent, with the tunneling time between sites above $10^{18}$ s. The number of lattice sites was typically between 5 to 35. The number of condensed atoms in each site could be varied between about 200 to $10^{4}$. Shaking the lattice as a whole, it is possible to create in each lattice site multimode states of excited Bose–Einstein condensates [95,96,97,98,119,120,121,122,123]. Such states are described by the statistical operator

(145) $${\hat{\rho }}_{n}\left(t\right)=\sum_{k=1}^{N_{M}}n_{k}\left(t\right)\;|\;kk\ldots k\;\rangle \langle \;kk\ldots k\;|\;,$$

where $n_{k}$ is the fraction of atoms in the *k*-th mode, $N_{M}$ is the number of modes in a lattice site, whose number is $N_{L}$. The normalization of the statistical operator to one, requires the validity of the summation

$$\sum_{k=1}^{N_{M}}n_{k}=1\;.$$

The entanglement-production is quantified by the measure

(146) $$\epsilon (\hat{\rho }\left(t\right))=\left(1-N_{L}\right)\log\;\underset{k}{\sup}\;n_{k}\left(t\right)$$

varying in the range

(147) $$0\leq \epsilon (\hat{\rho }\left(t\right))\leq \left(N_{L}-1\right)\log N_{M}\;.$$

The temporal evolution of the measure was studied for two and three modes [124,125,126,127].

For the realization of the multitrap multimode states it is important that the traps be identical, so that the transition frequencies between the ground-state condensate and the generated coherent mode be the same in all traps. Only then it is feasible to shake the lattice with a frequency that would be in resonance with the transition frequency of all traps, which is necessary for the generation of the same mode in these traps. A collection of different traps, such as effective potential wells in a random matter, where the so-called Bose glass can arise [128,129,130] is not appropriate, since these effective wells are not identical, hence are characterised by different transition frequencies.

### 13.2. Entangling by Evolution Operators

Entanglement production by the evolution operator

(148) $$\hat{U}\left(t\right)=e^{-iHt}$$

was studied in Refs. [46,131]. The operator norm was defined as the Hilbert–Schmidt norm. Calculations were performed for finite-site Heisenberg and Ising Hamiltonians. Depending on the system parameters, the evolution of the entanglement-production measure

(149) $$\epsilon (\hat{U}\left(t\right))=\log\;\frac{||\;\hat{U}\left(t\right)\;\left|\right|}{||\;{\hat{U}}^{⊗}\left(t\right)\;\left|\right|}$$

is periodic or quasiperiodic.

## 14. Coherence Phenomena

Coherence phenomena occurring in nonequilibrium systems can be treated as a kind of dynamic phase transitions. Therefore the temporal behavior of these coherent phenomena can also be accompanied by drastic changes in the order indices and entanglement production. Examples of these phenomena are given by the dynamics of strongly nonequilibrium spin systems [132,133] and radiating systems [134,135,136]. Being many-particle collections, these systems allow for considering order indices and entanglement production characterizing their temporal behavior. To describe nonequilibrium processes in these systems, it is convenient to resort to quasispin representation [134,135,136] based on the quasispin operators

$$S_{j}^{z}=\frac{1}{2}\;\sigma_{j}^{z}\;,\;S_{j}^{\pm }=\frac{1}{2}\;\sigma_{j}^{\pm }\;,$$

where the index *j* enumerates particles, or spins, and $\sigma_{j}^{\alpha }$ are Pauli matrices. The main observable quantities are the fractional population imbalance (or longitudinal spin polarization)

(150) $$s\equiv \frac{2}{N}\sum_{j=1}^{N}\langle \;S_{j}^{z}\;\rangle$$

and the dimensionless coherence intensity or radiation intensity

(151) $$w\equiv \frac{4}{N^{2}}\sum_{i\neq j}^{N}\langle \;S_{i}^{+}S_{j}^{-}\;\rangle$$

that are functions of time coming from the equations of motion. Summation is over all atoms or spins. It is useful to mention that in nonequilibrium processes, such as radiation, the population imbalance *s* is never identically equals minus one, which would mean an equilibrium state, when the coherence intensity *w* would be zero.

Since there are two physically different operators, one related to coherence intensity and the other describing the atomic population imbalance (spin polarization), it is convenient to consider two different types of correlation functions [137]. Dealing with the population-imbalance operators requires to define correlation functions using the *z*-spin operators. Thus, it is possible to introduce the first-order correlation operator

(152) $${\hat{Q}}_{1}=\left[\;Q_{ij}\;\right]\;,$$

with the matrix elements

(153) $$Q_{ij}\equiv \langle \;S_{i}^{z}S_{j}^{z}\;\rangle \;.$$

The second-order correlation operator is

(154) $${\hat{Q}}_{2}=\left[\;Q_{ijmn}\;\right]\;,$$

having the matrix elements

(155) $$Q_{ijmn}\equiv \langle \;S_{i}^{z}S_{j}^{z}S_{m}^{z}S_{n}^{z}\;\rangle \;.$$

Similarly, it is straightforward to define the correlation operators composed of the ladder spin operators, the first order

(156) $${\hat{R}}_{1}=\left[\;R_{ij}\;\right]\;,$$

with the elements

(157) $$R_{ij}\equiv \langle \;S_{i}^{+}S_{j}^{-}\;\rangle \;,$$

and the second order

(158) $${\hat{R}}_{2}=\left[\;R_{ijmn}\;\right]\;,$$

with the matrix elements

(159) $$R_{ijmn}\equiv \langle \;S_{i}^{+}S_{j}^{+}S_{m}^{-}S_{n}^{-}\;\rangle \;.$$

To calculate the traces and norms of the correlation operators, we shall need the following equalities for coinciding locations,

$$S_{j}^{+}S_{j}^{+}=S_{j}^{-}S_{j}^{-}=0\;\;S_{j}^{z}S_{j}^{z}=\frac{1}{4}\;,\;S_{j}^{+}S_{j}^{-}=\frac{1}{2}+S_{j}^{z}\;,\;S_{j}^{-}S_{j}^{+}=\frac{1}{2}-S_{j}^{z}\;,\;S_{j}^{+}S_{j}^{z}=-\;\frac{1}{2}\;S_{j}^{+}\;,\;S_{j}^{-}S_{j}^{z}=\frac{1}{2}\;S_{j}^{-}\;,\;S_{j}^{z}S_{j}^{+}=\frac{1}{2}\;S_{j}^{+}\;,\;S_{j}^{z}S_{j}^{-}=-\;\frac{1}{2}\;S_{j}^{-}\;.$$

Let us start with the operators ${\hat{Q}}_{n}$. For the traces, we have

(160) $$\mathrm{Tr}{\hat{Q}}_{1}=\sum_{j=1}^{N}Q_{jj}=\frac{N}{4}\;,\;\mathrm{Tr}{\hat{Q}}_{2}=\sum_{jn}Q_{jnnj}=\frac{N^{2}}{16}\;.$$

The partially traced operators

(161) $${\hat{Q}}_{1}^{\left(\alpha \right)}={\mathrm{Tr}}_{H/H_{\alpha }}{\hat{Q}}_{2}=\frac{N}{4}\;{\hat{Q}}_{1}\;\left(\alpha =1,2\right)\;\;$$

give the related nonentangling factor operator

(162) $${\hat{Q}}_{1}^{⊗}=\frac{Q_{1}^{\left(1\right)}⨂Q_{1}^{\left(2\right)}}{\mathrm{Tr}{\hat{Q}}_{2}}={\hat{Q}}_{1}⨂{\hat{Q}}_{2}\;.$$

For the norms, we find

(163) $$||\;{\hat{Q}}_{1}\;\left|\right|=\frac{1}{4}\;\left(1+Ns^{2}\right)\;,\;||\;{\hat{Q}}_{2}\;\left|\right|=\frac{1}{8}+\frac{N}{4}\;s^{2}+\frac{N^{2}}{16}\;s^{4}\;,\;||\;{\hat{Q}}_{2}^{⊗}\;||=||\;{\hat{Q}}_{1}\;{\left|\right|}^{2}=\frac{1}{16}\;{\left(1+Ns^{2}\right)}^{2}\;.$$

Calculating the order indices and entanglement-production measure, we simplify the final expressions by keeping in mind a large number of atoms N≫1. Then we get the order indices

(164) $$\begin{array}{c} \omega \left({\hat{Q}}_{1}\right)=\omega \left({\hat{Q}}_{2}\right)=\left\{\begin{array}{cc} 1,\; & \;s\neq 0\\ 0,\; & \;s=0 \end{array}\right. \end{array}$$

and the entanglement-production measure

(165) $$\begin{array}{c} \epsilon ({\hat{Q}}_{2})=\left\{\begin{array}{cc} 0,\; & \;s\neq 0\\ \log2,\; & \;s=0 \end{array}\right.\;. \end{array}$$

The behavior of the order indices here is similar to the case of magnetic systems. However the physical nature of the arising order is rather different from the latter. In the process of coherent dynamics or radiation, the ordering occurs for a short time, since s=s(t) is a function of time. Here it is an example of a dynamical order, while in a magnet, it is a stationary order. Similarly to the stationary magnetic order, the dynamic order also reduces the measure of entanglement production.

For the correlation operators related to the coherence intensity, we have the traces

(166) $$\mathrm{Tr}{\hat{R}}_{1}=\sum_{j}\langle \;S_{j}^{+}S_{j}^{-}\;\rangle =\frac{N}{2}\;\left(1+s\right)\;,\;\mathrm{Tr}{\hat{R}}_{2}=\frac{N^{2}}{4}\;{\left(1+s\right)}^{2}\;.$$

The partially traced operators

(167) $${\hat{R}}_{1}^{\left(\alpha \right)}=\frac{N}{2}\;\left(1+s\right){\hat{R}}_{1}\;\left(\alpha =1,2\right)$$

result in the nonentangling factor operator

(168) $${\hat{R}}_{2}^{⊗}=\frac{{\hat{R}}_{1}^{\left(1\right)}⨂{\hat{R}}_{1}^{\left(2\right)}}{\mathrm{Tr}{\hat{R}}_{2}}={\hat{R}}_{1}⨂{\hat{R}}_{1}\;.$$

We find the following norms

(169) $$||\;{\hat{R}}_{1}\;||=\frac{1}{2}\;\left(1+s\right)+\frac{N}{4}\;w\;,\;||\;{\hat{R}}_{1}^{\left(\alpha \right)}\;||=\frac{N}{2}\;\left(1+s\right)||\;{\hat{R}}_{1}\;\left|\right|\;,\;||\;{\hat{R}}_{2}^{⊗}\;||=||\;{\hat{R}}_{1}{\;||}^{2}\;,\;||\;{\hat{R}}_{2}\;\left|\right|=\frac{1}{2}\;{\left(1+s\right)}^{2}+\frac{N}{2}\;\left(1+s\right)w+\frac{N^{2}}{16}\;w^{2}\;.$$

Thus, for large N≫1, we obtain the order indices

(170) $$\begin{array}{c} \omega \left({\hat{R}}_{1}\right)=\omega \left({\hat{R}}_{2}\right)=\left\{\begin{array}{cc} 1,\; & \;w\neq 0\\ 0,\; & \;w=0 \end{array}\right. \end{array}$$

and the entanglement-production measure

(171) $$\begin{array}{c} \epsilon ({\hat{R}}_{2})=\left\{\begin{array}{cc} 0,\; & \;w\neq 0\\ \log2,\; & \;w=0 \end{array}\right.\;. \end{array}$$

These quantities essentially depend on the coherence intensity w=w(t) that plays here the role of a dynamic order characteristic. A nonzero *w* signifies the appearance of a coherent dynamic order. Sometimes, the occurrence of the coherent dynamic order is paralleled with the nonequilibrium magnon condensation [138,139,140]. Note that in equilibrium systems magnons cannot condense [141,142].

## 15. Conclusion

We have surveyed the meaning and applications of two concepts, order indices and entanglement production. The concept of order indices generalizes that of order parameters. The latter usually describe long-range order and are well defined only for macroscopic systems in the thermodynamic limit. However the order indices can be defined for any system, whether in the thermodynamic limit or finite. They characterize all types of order, long-range, mid-range, or short-range, making it possible not merely distinguishing the qualitative types of orders, but prescribing a measure uniquely quantifying the level of ordering and being applicable to equilibrium as well as to nonequilibrium systems.

The entanglement production describes how much entanglement is produced by an operator. This should be distinguished from the notion of entanglement describing the operator structure. It is possible to say that entanglement, characterizing the operator structure, is a static notion, while the entanglement production, describing the operator action, is a kind of a dynamic notion. The entanglement production can be quantified by a measure that is valid for any system, bipartite or multipartite, equilibrium or not.

It turns out that order indices and the entanglement-production measure are closely related with each other. As a rule, the larger the level of order in a system, the smaller the entanglement-production measure. The use of these concepts helps to better understand the properties of the studied systems and to more efficiently employ them in applications.

In the review, we considered the cases that could be treated analytically. Dealing with finite systems, it is usually necessary to resort to numerical calculations. Thus powerful numerical methods have been developed for studying the dynamics of finite Bose systems [143,144,145]. Hopefully, the described notions could be successfully employed for the analysis of finite systems by applying numerical methods.
